# Implementing Age-Friendly Hospital Initiatives: The MEITAV Model - A Synergistic Bottom-Up-Top-Down Approach

**DOI:** 10.1093/geroni/igaf122.525

**Published:** 2025-12-31

**Authors:** Ayelet Dagan Yarkoni, Natella Belinson, Orit Shahar, Rony Schenker, Adar Klein Druyan, Ruty Ofir, Anna Zisberg

**Affiliations:** JDC, Jerusalem, Yerushalayim, Israel; Ministry of Health, Jerusalem, Yerushalayim, Israel; JDC, Jerusalem, Yerushalayim, Israel; JDC, Jerusalem, Yerushalayim, Israel; Ministry of Health, Jerusalem, Yerushalayim, Israel; Ministry of Health, Jerusalem, Yerushalayim, Israel; University of Haifa, Haifa, HaZafon, Israel

## Abstract

The unprecedented demographic shift toward an aging population necessitates integrating age-friendly principles into hospital operations. This presentation examines MEITAV (Preventing Functional Decline of Older Adults During Hospitalization), an innovative implementation model combining leadership-driven strategies with grassroots initiatives to achieve sustainable hospital transformation. MEITAV represents a pioneering collaboration between government ministries and a civil society organization, creating a scalable framework implemented across all general hospitals in Israel. Our analysis demonstrates how strategic alignment of interdisciplinary teams, staff empowerment, and executive leadership has enhanced application of the 4Ms Framework—particularly mobility and mentation (Delirium)—while strengthening geriatric consultation services. Critical success factors include targeted professional development, environmental modifications, and evidence-based protocols. Initial implementation data shows engagement across 100 internal and surgical departments in 27 general hospitals, led by 120 multidisciplinary champions. Notably, 50% of hospitals meet mobility maintenance and improvement during hospitalization, while 76% achieve delirium screening objective for patients aged 75+. This presentation provides evidence-based insights into challenges, opportunities, and best practices from the MEITAV experience, offering valuable guidance for transforming isolated age-friendly initiatives into comprehensive institutional change, Initial clinical and economic outcomes will be presented, demonstrating MEITAV’s impact on improving care for hospitalized older adults

